# Current research in perineural invasion of cholangiocarcinoma

**DOI:** 10.1186/1756-9966-29-24

**Published:** 2010-03-10

**Authors:** Fang-Zhen Shen, Bing-Yuan Zhang, Yu-Jie Feng, Zhuo-Xia Jia, Bing An, Chang-Chang Liu, Xi-Yun Deng, Anil D Kulkarni, Yun Lu

**Affiliations:** 1 Department of Oncology, Affiliated Hospital of Medical College, Qingdao University, No 16 Jiangsu Rd, Qingdao 266003, China; 2 Second Department of General Surgery, Affiliated Hospital of Medical College, Qingdao University, No 16 Jiangsu Rd, Qingdao 266003, China; 3 Department of Bio-Information, Affiliated Hospital of Medical College, Qingdao University, No 16 Jiangsu Rd, Qingdao 266003, China; 4 Department of English Teaching, Shandong Medical College, Jinan 250002, China; 5 Animal Science Laboratory, Affiliated Hospital of Medical College, Qingdao University, No 16 Jiangsu Rd, Qingdao 266003, China; 6 Department of Surgery, University of Texas Health Science Center at Houston, 6431 Fannin Street, Houston, TX 77030, USA

## Abstract

**Background:**

Perineural invasion is a common path for cholangiocarcinoma (CCA) metastasis, and it is highly correlated with postoperative recurrence and poor prognosis. It is often an early event in a disease that is commonly diagnosed in advanced stages, and thus it could offer a timely therapeutic and diagnostic target if better understood. This article systematically reviews the progress of CCA neural invasion-related molecules.

**Methods:**

Studies were identified by searching MEDLINE and PubMed databases for articles from January 1990 to December 2009, using the keywords "cholangiocarcinoma," "perineural invasion," "nerve growth factor"(NGF), "neural cell adhesion molecule" (NCAM), "matrix metalloproteinase"(MMP), "neurotransmitter," "acetylcholine" (Ach), and "transforming growth factor" (TGF)." Additional papers and book chapters were identified by a manual search of references from the key articles.

**Results:**

From above we found that the molecules NGF, NCAM, MMP, Ach and TGF may have prognostic significance in, and offer clues to the mechanism of CCA neural invasion.

**Conclusions:**

Cholangiocarcinoma's increasing worldwide incidence is especially poignant in view of both the lacking effective therapies, and the fact that it is commonly diagnosed in advanced stages. As CCA neural invasion often appears early, more complete characterization of its molecular pathology could lead to the identification of targets for the diagnosis and therapy of this devastating malignancy.

## Review

Cholangiocarcinoma (CCA) is a malignant tumor originating from biliary tract epithelial cells. Among primary liver tumors, CCA incidence is only less than that of liver cancer[1,2], and it is becoming the most common hepatic tumor-induced death[3].

Due to its difficulty of diagnosis and high fatality rate, cholangiocarcinoma is extremely destructive, currently surgery is the only therapeutic mode offering a cure. Moreover, the post-resection recurrence rate is extremely high and the five-year survival rate is only 5%, at the same time, this survival rate had not vastly improved in past three decades[4]. In recent years, its worldwide morbidity and mortality have increased rapidly. Invasion delitescence, insufficient markers for early diagnosis marker, insensitivity to regular radio- and chemotherapy--these are all causes of poor prognoses of CCA patients[5,6].

Cholangiocarcinoma via perineural invasion is an extremely part during its genesis and development especially the early period. Perineural invasion (PNI) involves tumor cells surrounding nerve fibers, and entering the perineurium, spreading local infiltration and metastasis. The peripheral nerve is covered by three layers of membrane--the adventitia, perineurium and endomembrane. Carcinoma cells found in the perineurium are indicative of neural invasion[7]; the proportion of perineural invasion in CCA is around 85-88%. While the tumor perineural invasion is generated in cholangiocarcinoma, it indicated that the tumor is not only localized in the primary organ, but metastasis in distance or the tumor cell residue stays in abdominal cavity; furthermore, it is quite hard to radical cure by the operation and the clinical prognosis is extremely bad[8]. A study of 26 cases of neural invasion (NI) of CCA in the porta hepatis region revealed that the incidence of neural invasion was 100%. Survival rates of CCA patients without NI are clearly longer than those with NI, which indicates that the neural invasion is a common pathology for CCA--one that is highly correlated with postoperative recurrence and poor prognosis[9]. Some prognosis of perineural invasion was evidently less than that of non invasion patients, especially the tumor has not invaded into plasma membrane, nevertheless, while accompanied by tumor perineural invasion, the prognosis was even worse[10]. Therefore, PNI and postoperative recurrence rate are closely related. Consequently, if the mechanism of CCA PNI could be understood and interrupted in early-stage CCA, the prognosis of CCA patients could be greatly improved.

### Anatomic Foundation of Cholangiocarcinoma PNI

In the human hepatoduodenal ligament, the pampiniform nerve plexus can be clearly seen, and it can be classified into hepatic anteplex and hepatic metaplex. The hepatic anteplex is composed of the left and right celiac ganglia and left vagus nervous ramification, which includes the cystic duct, gallbladder and cholo-pancreatic common bile duct ramification. The scabbard is formed around the hepatic artery, and leads, via the hepatic artery, into the liver. The hepatic metaplex is composed of the right celiac ganglia and right vagus nerve ramification, which are mainly distributed along the extrahepatic bile duct and portal vein; some of its ramification links with the anteplex nervous ramification. The sensory fibers of the right phrenic nerve are distributed in the coronary ligament, the falciform ligament of the liver, and the vicinal liver capsule[11], while part of the fibers combined with the liver ante- and metaplex, along with the fibers of the hepatic plexus, and distributes into the exterior and interior biliary system of the liver. The whole liver is controlled by the sympathetic and parasympathetic nerves. They are distributed in the hepatic artery, vena portae hepatic, liver interior and extrahepatic bile duct; the sympathetic nerve originates from celiac ganglia, while the parasympathetic nerve comes from the vagus nerve[12]. Therefore, the biliary system is typical of organs with extremely fundamental autonomic nerves, which could be controlled by an extensive neural system. The nerve terminal is partially removed through the porta hepatic hemal tube structure, surrounded by the bile duct and blood vessel. The bile duct is one of the most important components of the liver, which is also the channel of choleresis and excretion. As the nerve terminal acts on the liver hemal tube system, the patho- and physiological functions of bile duct epithelium are inevitably affected, providing the anatomic foundation for CCA metastasis via PNI.

### Cholangiocarcinoma PNI as independent metastasis pathway

Among gastrointestinal malignancies, PNI is often seen in pancreatic and biliary system cancers, and occasionally in rectal cancer. It is a local diffusion mode for tumors, and it plays a critical role in prognosis. Current study shows tumor perineural invasion to be uncorrelated with patient's age or sex as well as whether or not tumor metastasis in distant (including liver metastasis or abdominal cavity, peritoneum metastasis). However, it is highly correlated with tumor volume, location, depth of invasiveness, angiogenesis and lymph node involvement[13]. After CCA saturated the whole cliff, nearly hundred percent of tumor occurred nerve infiltration, which was far higher than lymphatic metastasis and circulatory metastasis.

Physically, the biliary system is close to both the peripheral nerve plexus and the coelial plexus, which proximity may facilitate peripheral nerve invasion by biliary tumors. Some reports consider that the biliary system is rich in autonomic nerves, which may also facilitate perineural invasion[14]. However, neither of these facts completely explains the specific mechanism of tumor cells entering into nerve tissue. Recent investigation has indicated that the relationship between PNI occurrence and the distance between tumor and nerve plexus was not close. Secondly, the tumor cells invade nerves via the perineural lymphatic vessel. Previous studies considered that tumors invade nerves along the "path of least resistance," or are transported along blood and lymphatic pathways[15,16]. However, in rectal cancer, especially distal rectal cancer, although these tumors are close to the sacral nerve plexus, one study found that the rate of perineural invasion is rather low, only 9.9-34.9% [17]; this investigation also indicated that nervous invasion was not correlated with the location of carcinoma swelling, volume, histology category, at even the status of lymphatic metastasis. Tumors had previously been thought to invade nerve through the lymphatic pathway in the nerve or perineurium. However, an investigation found that about 34% of pancreatic carcinoma patients with NI were without lymphatic metastasis, while 75% of such patients without any NI appeared to have lymphatic metastasis. Therefore, it is considered that the possibility of the patients with widespread lymphatic metastasis who emerged peripancreatic nervous invasion was quite high. However, peripancreatic nervous invasion is not completely determined by lymphatic pathway. Another report found no perineural lymphatic vessel, by either electron microscope or light microscope; however, they found that nerves in the perineurium can be separated from their peripheral connective tissue, generating low-resistance, slit-like interspaces in the nerve periphery, which are easily invaded by tumor cells[18], which suggests that if a tumor came through perineural lymphatic vessel, then the nerve environment could be a focus of jump infection with lymphatic metastasis characteristics. Moreover, the tumor will not offend the nerve for a wrap. If tumor cells invade nerves through the low-resistance perineural layer, then the insufficiency of the leap focus of infection was bound to invade the nerve for a wrap. So the femoral nerve of the rats and Walk2er256 tumor cell were incubated together by Rodin, one week later, the tumor cells completely wrapped the nerve and without any leap focus of infection. Recent progressive investigation also found that the perineurium was available in three different weak positions. Such as entrance and exit of blood vessel, invasion court of reticular fiber. The pancreatic cancer invaded into perineurium through its weak part, then spread and diffused along the perineural interspace to the outside of the perineurium, forming a new metastasis, which was not the previous presumed lymphatic diffusion pathway. Meanwhile, from the research on esophageal carcinoma, a report also revealed that the tumor cell infiltrated periphery nerve was not accorded with cell of lymphatic glands[19]. Consequently, it was impossible that the tumor cell invaded peripheral nerve tissue through peripheral lymphatic vessels, nor was any direct relationship involved in the tumor peripheral nerve infiltration and lymphatic metastasis.

Another study reestablished modes of CCA nervous invasion and metastasis using computer-assisted three-dimensional (3D) reconstruction. The computer-formed CCA 3D stereoscopic pictures, showing the spatial relationships between CCA and nerves, lymphatic vessels and blood vessels, revealed that small vessels, lymphatic vessel and nerve fibers all existed in the tumor periphery, offering an anatomic foundation for CCA nerve invasion. In particular, the 3D CCA model showed that tumor cells in the nervous peripheral interspace are able to survive independently, as they are in small blood and lymphatic vessels[20]. All the above investigations indicate that tumor perineural invasion is actually a type of tumor local growth pattern. The perineural interspace invasion was the fifth dependent metastasis pathway to be discovered (precededafter abdominal tumor direct invasion metastasis, implantation metastasis, lymphatic, and blood route metastasis). In PNI, leap metastasis is possible; e.g., CCA could metastasize into liver via the neural interspace.

## Progress of Cholangiocarcinoma PNI-related Molecules

### Effect of NGF on CCA PNI

Nerve growth factor (NGF) was the first discovered member of the neurotrophic factor family; this family is widely expressed in tumor tissues, and is involved in tumorigenesis and tumor growth. Receptors for NGF include two different proteins: TrkA, which has high affinity, and is a Tyr protein kinase receptor encoded by the proto-oncogene trk; and NGF receptor p75, which has low affinity. The protein p75 is a glycoprotein mainly expressed in NGF-reactive cells; it is involved with apoptosis and cell migration[21]. One report, using the bile duct ligation model, showed NGF and its receptor TrkA to be expressed in common bile duct epithelium[22] They also discovered the proliferative response of fibroblase, elastic fiber in bile duct connective tissue, accompanied by elevated expression of NGF and its receptor TrkA. This indicates that NGF and TrkA both play critical roles in the proliferation of connective tissue in the bile duct. Some experimental results proved that NGF expressed in CCA could facilitate CCA cell proliferation[23,24]; while NGF secreted by CCA cells could facilitate autospecific proliferation, it might also bind to TrkA receptors expressed in the perineurium, which supplies an adaptive microenvironment and chemical tropisms for the growth of nerve cellular axons, facilitating axon growth in the direction of the tumor[25]. The expression of NGF and its receptors in a wide range of tumor cells show its critical relationship with tumor proliferation and invasion, especially in nerve tissue. So its signal pathway was able to be used as the target for the early intervention and therapy.

### Effect of Neural Cell Adhesion Molecules on CCA PNI

Neural cell adhesion molecules (NCAM) belong to the adhesion molecule immunoglobulin family, which belongs to IgG super family and mediates cellular adhesion. NCAMs play critical navigation and docking roles by binding to target cells during the growth and development of the nervous system. NCAM is highly expressed in peripheral nerve tissue. It has an ecotropic relationship to nervous tissue and plays a critical role in the genesis and metastasis of CCA[26]. Some researches found that NI is correlated with NCAM expression, indicating that NCAM molecules on the surface of tumor cells might induce them to migrate and adhere to nerve cells after the tumors breach their capsules[27]. In particular, NCAM expression is highly correlated with CCA PNI, and with CCA dedifferentiation. Moreover, NCAM was shown to be a specific indicator for bile duct NI. A study of the relationship between the expression of NCAM and the anti-oncogene DPC4, and CCA NI, showed that the NCAM expression rate in CCA with NI was significantly higher than in CCA without NI, indicating that NCAM is related to CCA NI and might play a critical role in the nerve invasion process[28]. NCAM expression rates generally increase with CCA invasiveness, indicating a relationship between NCAM expression and cancer cells' ability to adhere to nerve tissue, thus enabling nervous invasion. Recent evidence indicates that activation of the proto-oncogene K-Ras in pancreatic cancer cells could induce the up-regulation of PSA-NCAM on tumor cell surfaces. PSA-NCAM could bind to N-cadherin, blocking N-cadherin mediated cell adhesion, increasing pancreatic cancer cell migration ability and facilitating tumor cell metastasis to nerve tissue[29]. The above investigations all suggest that NCAM levels are positively correlated to CCA NI, and which might serve as indicators for prognosis in CCA.

### Effect of Matrix Metalloproteinases on CCA PNI

Matrix metalloproteinases (MMPs) are a family of zinc finger-dependent endogenous proteinases. Previous investigation showed MMPs to be critical enzymes which are able to decrease ECM, in addition, it was a specific growth factor (for instance, ECM related growth factor) hard to diffuse in the activation of ECM or hidden by matrix, so which that facilitate the tumor cells through the basement membrane. MMPs are involved in multiple cancer-related processes such as tumorigenesis, growth, migration, angiogenesis and anti-apoptotic functions[30,31]. Some reports investigating the correlation between S100, MMP-7 and CCA nerve invasion have found that expression of MMP-7 in CCA tissue is higher than in latero-carcinoma tissue; moreover, it shows significant correlation with the tumor lymphatic metastasis[32,33], Which indicates that MMP protein might be involved in CCA oncogenesis, and its up-regulation may facilitate CCA metastasis, and which further suggests that activation of MMP-7 is a critical promoting agent of CCA NI; high MMP-7 expression seems to correlate with higher malignancy and less favorable prognosis in CCA sufferers.

Reportedly, MMP-9 secretion is significantly enhanced in CCA cells that invade nerve tissue; it has been suggested that some component in peripheral nerves is able to induce MMP-9 secretion in CCA cells[34]. A novel signaling pathway of MMP-9 up-regulation in CCA cells has been proposed that features TNF-alpha-induced activation of COX-2 and PGE2 via TNF-R1, could be followed by up-regulation of MMP-9 via the PGE2 (EP2/4) receptor[35]. Recent reports indicate that corpora mammillaria CCA, which is less prone to PNI than most CCA, is characterized by comparatively low expression of MT-MMPs, as well as better prognoses[36]. For this reason, MMPs expression is a critical reference index for assessing CCA bionomics and the evaluation of prognosis.

### Effect of Neurotransmitters on CCA PNI

#### Sympathetic nervous system

The first clue to the role of the sympathetic nervous system in regulating CCA growth was the discovery that the α-2A, α-2B, and α-2C adrenergic receptor subtypes were all expressed in the CCA cell lines Mz-ChA-1 and TFK1. In a further investigation, after applying α-2 adrenergic receptor agonist, uK14, they found that uK14 could inhibit the growth of CCA by stimulating tumor cells[37]. Recent evidence revealed that expressions of α-1 adrenergic receptor and β-2 in CCA cells that generate peripheral nervous metastasis and lymphatic metastasis were significantly higher than in non-metastatic CCA cells[38]. In addition, NE could facilitate the cell proliferation and metastasis of CCA, while applying the relative receptor blocker might significantly inhibit this kind of promotion. The CCA environment is regionally rich in sympathetic nerve fibers, offering the sort of intercommunication conducive to perineural invasion. This mechanism needs some further investigations.

#### Parasympathetic Nervous System

The parasympathetic nervous system (PSNS) plays a critical role in the oncogenesis of bile duct cells. The main neurotransmitter secreted by PSNS is acetylcholine (Ach), which has been shown to mediate cellular transformation and differentiation[39], and might play a critical role in normal cellular proliferation, differentiation, transformation, as well as tumorigenesis etc[40]. Multiple experiments have confirmed Ach expression in various tumors, notably metastatic small-cell lung cancer[41]. It appears that Ach is involved in diseases far beyond its effects as a neurotransmitter. Previous evidence showed that the muscarinic acetylcholine receptor is expressed in the gallbladder cancer cell line Mz-ChA-1; while cultivating the muscarinic AchR receptor agonist carbonic acid bilineurin with the gallbladder cancer cells, the activation of the IP3 signal and enhancement of calcium level were observed[42]. Other research assumed that, with the stimulation of different molecules, IP3 and calcium level played critical roles in the inhibition of CCA growth. However, muscarinic AchR is directly activated by other molecules; bile acid has been found to stimulate M3 AchR, a reaction mediated by EGFR, thus stimulating the proliferation of colon carcinoma cells[43]. This kind of effect could induce the phosphorylation of p10RSK via the Ca/MEK/MAPK dependent pathway. Some reports showed that Ach could up-regulate expression of DNA repairase PRX1 and promote cell differentiation in lung cancer, for which a possible correlation between Ach and cancer cell transformation has been indicated[44,45]. However, the role of PSNS with regard to CCA-PNI has currently not been elucidated; considering the critical regulatory effect of the vagus nerve on the biliary system, it is likely that the PSNS plays a regulating role in CCA-PNI.

### Effect of TGF on CCA PNI

In 1980s, investigators found that some tumor cells could produce a polypeptide, transforming growth factor (TGF), which could stimulate inactive growth cells into activated growth cells. The polypeptide came into two types, TGF-α and TGF-β. Previous investigation indicated that TGF-β1 was highly expressed in most tumor cells, and that over-expression of TGF-β in tumor was associated with tumor growth, metastasis, angiogenesis, and dedifferentiation[46]. High expression of TGF-β was also detected in colorectal cancer, gastric cancer, breast carcinoma, prostatic carcinoma, bladder carcinoma and endometrial cancer, and which was associated with tumor succession, growth and metastasis[47,48]. Tumor cell metastasis is a kind of reversible epithelium-to-mesochymal transformation (EMT) in vivo, this was possibly a transient differentiation event, in the anaphase of tumorigenesis, TGF-β directly affected the tumor cell and accelerated the growth of tumor. Then the activation of Akt/PKB was induced by TGF-β via RhoA and PI-3K pathway, subsequently, Z0-1 was activated, cell morphous altered, the cell-cell junction changed, and finally the tumor metastasis was induced.

Zhang et al found that[49], with the enhancement of CCA clinical stage, the expression of TGF-β1 increased, indicating that TGF-β1 could be involved in the genesis, growth and clinical scale of CCA, as well as perineural lymphatic invasion. Lu et al. also reported that TGF-β1 expression increased with tumor grade, suggesting that TGF-β1 not only suppresses growth but can also suppress immunity[50]. In HCCs, TGF-β1 expression is enhanced (compared to adjacent tissues), while TGF-βR2 expression is weakened, due to lower TGF-βR2 expression in those HCC cells that can escape from the inhibitory effects of TGF-β1. Some initiation and development of HCC may be caused by this process. In all MMTV-PyVmT tumor cells, the inhibition of TGF-β could significantly depress basal cell mobility, survival rate, anchoring dependent growth, tumorigenesis and metastasis, indicating that variations in metastasis are controlled by auto-regulation of epithelial cells[51]. Current reports show that the overexpression of TGF-α is common in gastrointestinal tumors. otherwise, generous animal studies confirmed that while the carcinomatous change was occurred, three different mode of action such as autocrine, paracrine and juxtacrine were all available, and autocrine circulation was the main mode for TGF-α. Zhuang et al[49]. showed that overexpression of TGF-α was common in CCA cells, suggesting a mechanism in which cytogenic TGF-α first binds to EGFR, which in turn activates tyrosine protein kinase (Tyr-PK) [52]. In fact, EGFR-activated Tyr-PK could facilitate DNA synthesis and cause cell proliferation and differentiation. Moreover, with the collective effect of other factors, a cell starting malignant transformation could secrete TGF-α, inducing hyperexpression of TGF-α and EGFR, and causing uncontrolled growth [53]. Either of these mutual effects could generate signals that facilitate cancer cell proliferation and growth, stimulating its diffusion and generating nervous invasion. Thus, TGF plays a critical role in the proliferation of digestive system tumors and NI, especially in CCA.

The proliferation of CCA through perineural invasion is a pathological process with multiple factors and processes. We aim to focus on its possible mechanisms, and search for novel methods and targets to prevent perineural invasion in early-phase CCA.

## Conclusions

Cholangiocarcinoma is difficult to diagnose; consequently it is commonly identified in its advanced and least treatable stages. However, CCA neural invasion often occurs early on, suggesting that more complete characterization of this pathway could help identify more timely therapeutic and diagnostic targets for this devastating malignancy.

## Abbreviations

(CCA): cholangiocarcinoma; (NGF): nerve growth factor; (NCAM): neural cell adhesion molecule; (MMP): matrix metalloproteinase; (Ach): acetylcholine; (TGF): transforming growth factor; (PNI): perineural invasion; (NI): neural invasion; (PSNS): parasympathetic nervous system.

## Funding

This work was supported by a grant from the Medical Academic Program of Qingdao City (No. 2009-WSZD073) and the Foundation of Most Advanced Group of Medical Scientists and Technicians of Shandong Province.

## Ethical approval

Not needed.

## Competing interests

No benefit in any form has been received or will be received from any commercial party related directly or indirectly to the subject of this article.

## Authors' contributions

ZBY and LY proposed the design of the study, SFZ and FYJ participated the main body of the article and drafted the manuscript. JZX and LCC have participated in the data in the study, AK, AB and DXY participated in its coordination and helped to draft the manuscript. LY is the guarantor. All authors read and approved the final manuscript.

## Authors' information

Fang-Zhen Shen, M.D. Department of Oncology, Affiliated Hospital of Medical College, Qingdao University, No.16 Jiangsu Rd, Qingdao 266003, China. E-mail fangzhenshen@126.com

Bing-Yuan Zhang, M.D. Second Department of General Surgery, Affiliated Hospital of Medical College, Qingdao University, No.16 Jiangsu Rd, Qingdao 266003, China. E-mail bingyuanzhang@126.com

Yu-Jie Feng, M.D. Second Department of General Surgery, Affiliated Hospital of Medical College, Qingdao University, No.16 Jiangsu Rd, Qingdao 266003, China. E-mail fengyj1943@163.com

Zhuo-Xia Jia, B.S. Department of Bio-Information, Affiliated Hospital of Medical College, Qingdao University, No.16 Jiangsu Rd, Qingdao 266003, China. E-mail xuhaoqd@gmail.com

Bing An. B.S. Department of English Teaching, Shandong Medical College, Jinan 250002, China. E-mail yatou_filly@sina.com

Chang-Chang Liu, B.S. Animal Science Laboratory, Affiliated Hospital of Medical College, Qingdao University, No.16 Jiangsu Rd, Qingdao 266003, China. E-mail: yatou@126.com

Xi-Yun Deng, M.D. & Ph.D. Department of Surgery, University of Texas Health Science Center at Houston, 6431 Fannin Street, Houston, TX 77030, USA E-mail: Xiyun.Deng@uth.tmc.edu

Anil D Kulkarni, MSc. & Ph.D. Department of Surgery, University of Texas Health Science Center at Houston, 6431 Fannin Street, Houston, TX 77030, USA E-mail: Anil.D.Kulkarni@uth.tmc.edu

Yun Lu, M.D. & Ph.D Second Department of General Surgery, Affiliated Hospital of Medical College, Qingdao University, No.16 Jiangsu Rd, Qingdao 266003, China. E-mail: luyunmd@gmail.com
